# Impact of Metformin therapy on *miR-9*, *miR-223*, and *miR-132* and inflammasome-related gen expression in obese and non-obes PCOS patients: A comparative study with healthy controls

**DOI:** 10.1371/journal.pone.0335280

**Published:** 2025-12-18

**Authors:** Seyed Alireza Mirjalili, Seyed Mehdi Kalantar, Fateme Montazeri, Reyhaneh Azizi, Elham Hosseini, Fateme Zare, Samira Asgharzade, Korosh Ashrafi Dehkordi

**Affiliations:** 1 Department of Molecular Medicine, School of Advanced Technologies, Shahrekord University of Medical Sciences, Shahrekord, Iran; 2 Abortion Research Center, Yazd Reproductive Sciences Institute, Shahid Sadoughi University of Medical Sciences, Yazd, Iran; 3 Diabetes Research Center, Shahid Sadoughi University of Medical Sciences, Yazd, Iran; 4 Department of Obstetrics and Gynecology, School of Medicine, Zanjan University of Medical Sciences, Zanjan, Iran; 5 Reproductive Immunology Research Center, Shahid Sadoughi University of Medical Sciences, Yazd, Iran; 6 Cellular and Molecular Research Center, Basic Health Sciences Institute, Shahrekord University of Medical Sciences, Shahrekord, Iran; Shiraz University of Medical Sciences, IRAN, ISLAMIC REPUBLIC OF

## Abstract

**Introduction:**

Polycystic Ovary Syndrome (PCOS) is a common endocrine and metabolic disorder characterized by chronic inflammation, insulin resistance, and hormonal imbalances, often leading to infertility and metabolic dysfunction. Metformin, an insulin-sensitizing agent, has shown potential to improve these conditions. This study investigated the impact of metformin on inflammasome-regulating microRNAs (*miR-9, miR-223, miR-132*) and related genes (*IL-1β, IL-18, caspase-1, NLRP3*) in obese and non-obese PCOS patients compared to healthy controls.

**Materials and methods:**

In this case-control study, 100 women aged 18–35 were divided into 50 PCOS patients and 50 controls, stratified by BMI (>25 kg/m² and <25 kg/m²). Blood samples were analyzed pre- and post-12 weeks of metformin treatment (500 mg twice daily) for serum hormone levels (FSH, LH, TSH) by ELISA kit, miRNA and mRNA expression by qPCR, and follicle count by transvaginal ultrasound were evaluated.

**Results:**

The results demonstrated a significantly lower expression of *miR-9* in PCOS patients (BMI >25 kg/m²) compared to healthy controls (mean ± SD: 0.54 ± 0.07 vs. 1.00 ± 0.11; *P* < 0.001). Following metformin treatment, *miR-223* expression was significantly upregulated (from 0.88 ± 0.06 to 1.21 ± 0.08; *P* = 0.002). Similarly, the expression levels of *IL-1β* (2.01 ± 0.31 vs. 1.31 ± 0.23) and *NLRP3* (2.12 ± 0.27 vs. 1.38 ± 0.22) decreased significantly post-treatment (P < 0.01). No significant change was observed in *miR-132* expression. Overall, metformin modulated the expression profiles of inflammasome-related genes and miRNAs, particularly in obese patients with PCOS.

**Conclusion:**

The findings suggest that metformin modulates inflammation in PCOS by altering microRNA and inflammasome-related gene expression, thereby reducing inflammatory markers such as IL-1β and miR-9, while enhancing miR-132 and miR-223, which may contribute to improved metabolic and inflammatory profiles. These results support the use of metformin as a BMI-tailored therapeutic strategy for PCOS, warranting further research to confirm its long-term effects and mechanisms.

## Introduction

Polycystic Ovary Syndrome (PCOS) is a common, complex, and heterogeneous endocrine and metabolic disorder affecting approximately 6% to 20% of women of reproductive age. Its development is significantly influenced by environmental factors such as diet, lifestyle, and socio-economic conditions [1–6]. The condition is often associated with symptoms like obesity, acne, hirsutism, and alopecia. Additionally, insulin resistance and hyperinsulinemia are frequently observed among PCOS patients [7,8], leading to complications such as infertility [9]. In recent years, insulin-lowering agents have been proposed as a potential therapeutic approach for PCOS. Among these, metformin, a biguanide with insulin-sensitizing properties, has been shown to reduce hyperinsulinemia and hyperandrogenemia levels, typical features of PCOS, thereby improving ovulation [10,11]. Metformin is well-known for decreasing insulin resistance by enhancing insulin receptor signaling, reducing circulating insulin levels, and normalizing the LH/FSH ratio, thus promoting follicular development and ovulation [12–14]. Additionally, it decreases androgen production, reduces symptoms of hyperandrogenism, stabilizes prolactin levels for more regular menstrual cycles, and lowers anti-Müllerian hormone (AMH) levels, indicating improved follicular dynamics [15–17].

The etiology of PCOS remains debatable, but inflammation is thought to play a key role. This includes abnormal ovarian tissue remodeling during folliculogenesis and elevated inflammatory markers such as C-reactive protein (CRP), which are associated with the condition [18,19]. Studies suggest that in overweight or obese women, visceral adipocytes release inflammatory cytokines like tumor necrosis factor α (TNF-α) and interleukin 6 (IL-6), which contribute to insulin resistance and activate the inflammasome pathway, thus exacerbating PCOS symptoms [19,20]. The inflammasome complex is essential in PCOS, particularly in the production of *IL-1β* and *IL-18*, which may contribute to improving the immunopathogenesis of PCOS. The inflammasome is a multi-protein complex within the cytoplasm that is assembled in response to various danger signals, such as uric acid crystals, reactive oxygen species (ROS), ATP, free fatty acids (FFA), high mobility group box 1 (HMGB1), heat-shock proteins, and pathogens. Upon activation, the inflammasome leads to the maturation of inflammatory cytokines like IL-1β and IL-18 through Caspase-1 activation [19,21,22].

The inflammasome is expressed in ovarian granulosa cells (GCs) and can become abnormally activated under pathological conditions or in response to various stimuli [23]. Its activation is regulated by different mechanisms, notably through post-transcriptional modulation by microRNAs (miRNAs) [24]. miRNAs are small, non-coding RNA molecules (~20–23 nucleotides) that regulate gene expression by targeting the 3ʹ untranslated regions (UTRs) of mRNAs [25]. They can repress translation or promote the degradation of target mRNAs, thereby playing crucial roles in cell functions such as migration, proliferation, and apoptosis. Dysregulation of miRNAs is associated with various diseases, including cancers [26], neurodegenerative diseases [27], and metabolic disorders [28]. Recent findings indicate that several miRNAs are abnormally expressed in PCOS patients [24,29,30].

Specifically, *miR-9* has been found to inhibit inflammasome activation by targeting the JAK/STAT signaling pathway [31]. *miR-223* can lower *IL-1β* levels by targeting the 3’UTR of the NLRP3 protein [32,33], while *miR-132* might inhibit inflammasome activation through the downregulation of FOXO3 via the FOXO3 signaling pathway [24].

This study aims to investigate the expression of these inflammasome-regulating miRNAs (*miR-9*, *miR-223*, and *miR-132*) pre- and post-treatment in obese PCOS patients, in comparison to healthy individuals. Based on previous research, these miRNAs might play vital roles in inflammation, insulin signaling, and previous associations with PCOS pathophysiology. Additionally, we will assess the levels of key inflammasome-related genes, including *IL-1β*, *Caspase-1*, *NLRP3*, and *IL-18*, to deepen our understanding of the role of inflammasome activation in PCOS pathogenesis.

## Materials and methods

### Study design

A total of 100 women, aged 18–35 years, were enrolled in the study, comprising 50 women diagnosed with PCOS based on the Rotterdam criteria [34] and 50 healthy controls. Participants were further categorized into two subgroups based on their body mass index (BMI classification: individuals with a BMI > 25 kg/m², categorized as overweight or obese, and those with a BMI < 25 kg/m², categorized as normal weight. Each BMI subgroup was then divided into two groups of 25 individuals each, consisting of patients and controls. Exclusion criteria included metabolic or endocrine disorders, infections, autoimmune diseases, anatomical abnormalities, and any history of maternal or paternal chromosomal abnormalities. The study received ethical approval from the Ethics Committee in Human Research under the code IR.SKUMS.REC.1401.100 and Grant number 6043. All patients provided written informed consent to participation in the study, confirming their voluntary involvement and understanding of the research procedures.

### Blood sampling

For blood sample collection, 10 mL of peripheral blood was obtained from each participant using EDTA-coated tubes. The collected blood was then divided into two aliquots: 5 mL for serum separation and 5 mL for the isolation of peripheral blood mononuclear cells (PBMCs). Serum was separated for subsequent biochemical and hormonal assays, while PBMCs were isolated using Ficoll density gradient centrifugation to ensure the purity of mononuclear cells. Both the serum and PBMC samples were stored at −80°C to maintain sample integrity for subsequent molecular analyses.

### Biochemical study, and the follicle count

The serum levels of LH, FSH, and TSH were determined on the third day of the menstrual cycle using a commercially available ELISA kit (Pishtaz Teb, Iran, catalog number), following the manufacturer’s instructions. Insulin and fasting blood glucose levels were measured using an auto-analyser. Additionally, ovarian morphology and the number of small antral follicles (often 2–9 mm in diameter) were assessed through transvaginal ultrasound using a high-resolution ultrasound device, performed by a trained radiologist to ensure accuracy and consistency in measurements.

### Molecular analysis

Total RNA was extracted from PBMCs using a commercial RNA isolation kit (Pars-Toos, Iran). The quality and concentration of extracted RNA were determined using a NanoDrop spectrophotometer (Eppendorf, Germany), ensuring an A260/A280 ratio between 1.8 and 2.0. The extracted RNA was then converted to complementary DNA (cDNA) using the BONmiR High Sensitivity MicroRNA 1st Strand cDNA Synthesis kit (Pars-Toos, Iran). Reverse Transcription Quantitative-PCR (RT-q-PCR) was performed to quantify the expression levels of *IL-1β*, *NLRP-3*, *Caspase-1, IL-18*, *miR-9*, *miR-223*, and *miR-132* using SYBR Green Master Mix (Ampliqon, Denmark) and a real-time -PCR system (Applied Biosystem, USA). Fold changes were calculated using the 2^-ΔΔCT^ method, with β-actin and U6-snRNA as internal controls for mRNA and miRNA, respectively. Moreover, primer sequences for mRNAs (IL-1β, NLRP3, Caspase-1, IL-18) and microRNAs (miR-9, miR-132, miR-223) are provided in Table 1.

**Table 1:** Primer sequences used for gene expression analysis.

Genes	Primer sequence (5´-3´)	Accession No. Homo sapiens	PCR product (bp)
***NLRP-3***	F: GGAGTGGATGGGTTTACTGGAG R: CGTGTGTAGCGTTTGTTGAGG	NM_001079821.3	165
***CASPASE-1***	F: TGAATACCAAGAACTGCCCAAG R: GCATCATCCTCAAACTCTTCTGTAG	NM_001257118.3	157
***IL-1β***	F: AGCTCGCCAGTGAAATGATG R: TGTAGTGGTGGTCGGAGATT	NM_000576.3	156
***IL-18***	F: TCTTCATTGACCAAGGAAATCGG R: TCCGGGGTGCATTATCTCTAC	NM_001386420.1	75
***B Actin***	F: CTGGAACGGTGAAGGTGACA R: AAGGGACTTCCTGTAACAATGCA	NM_001101.5	140
**hsa-mir-9-5p**	F: GCGGGTACTCTTTGGTTATCTAGC	MIMAT0000441	-
**hsa-mir-223-3p**	F: GGCTGGTGTCAGTTTGTCAA	MIMAT0000280	-
**hsa-mir-132-3p**	F: CCGAGGTAACAGTCTACAGCCA	MIMAT0000426	-
**snRNA-U6**	F: GTGCTCGCTTCGGCAGCA	-	-

### Treatment protocol

Metformin (500 mg twice daily) was administered to the PCOS group for 12 weeks. After the treatment period, blood samples were collected again from the patients, and the serum and PBMC samples were processed as described previously. The Serum levels of FBS, T3, T4, TSH, FSH, and LH, as well as the expression of target microRNAs, were re-evaluated. No dietary or lifestyle modifications were introduced during the study period.

### Statistical analysis

Statistical analysis was performed using GraphPad Prism 9.0 (GraphPad Software, USA). The values were presented as mean ± SD. One-way ANOVA followed by Tukey’s post-hoc test was used for comparison among more than three groups.. Furthermore, the relationship of quantitative variables was evaluated using Pearson’s correlation coefficient test.

## Result

### Demographics and clinical parameters of participants

Table 2 lists the demographic characteristics of study participants. In the PCOS group, the mean age was 33.3 ± 2.51 years for patients with BMI > 25 and 27.5 ± 5.05 years for those with BMI < 25. In the control group, the mean age was 31.38 years for individuals with BMI > 25, 29.87 years for those with BMI < 25, and 30.39 years overall.

**Table 2:** Demographic characteristics of study subjects.

Variables	Control	PCOS (Pre-treatment)	PCOS (Post-treatment)	P-Value
FBS (mg/dL)	87.35 ± 1.33	91.63 ± 2.23	88.79 ± 1.24	0.32 a,b: 0.71 a,c: 0.30 b,c: 0.58
T3 (mlu/L)	1.12 ± 0.04	1.44 ± 0.05	1.44 ± 0.05	0.0001 a,b: 0.81 a,c: 0.0002 b,c:0.0004
T4 (mlu/L)	6.79 ± 0.25	8.42 ± 0.28	8.43 ± 0.21	0.0001 a,b: 0.29 a,c: 0.0001 b,c:0.0001
TSH (mlu/L)	3.78 ± 1.53	3.50 ± 0.79	4.04 ± 1.22	0.10 a,b: 0.97 a,c: 0.15 b,c:0.10
FSH (mlu/L)	7.98 ± 1.28	8.41 ± 1.67	6.85 ± 2.12	0.008 a,b: 0.03 a,c: 0.9 b,c: 0.02
LH (mlu/L)	10.21 ± 1.76	13.61 ± 1.40	11.27 ± 1.47	0.006 a,b: 0.029 a,c: 0.03 b,c: 0.21

Data expressed as means± SD.

Data are statistically significant at *p* < 0.05.

a,b: Comparison between Control and PCOS Pre-treatment .

a,c: Comparison between Control and PCOS Post-treatment.

b,c: Comparison between PCOS Pre-treatment and PCOS Post-treatment.

### Comparison of serum level of biochemical parameters

Hormonal testing in patients indicated potential changes in hormone levels. FSH levels were lower in the metformin-treated PCOS group compared to the untreated PCOS and control groups, with a significant reduction observed during post-metformin treatment relative to pre-treatment levels (P = 0.03). However, other hormonal differences between groups were not statistically significant (P > 0.05). LH levels were significantly elevated in the PCOS group Pre-treatment compared to both the metformin-treated PCOS and control groups (P < 0.05). Additionally, T3 and T4 levels were significantly lower in the control group compared to PCOS patients, both pre and post treatment (P < 0.001). No significant differences were observed in FBS or TSH levels among the three groups (P > 0.05) (Table 2).

### Comparison of the expression level of miRNAs

**Fig 1:**
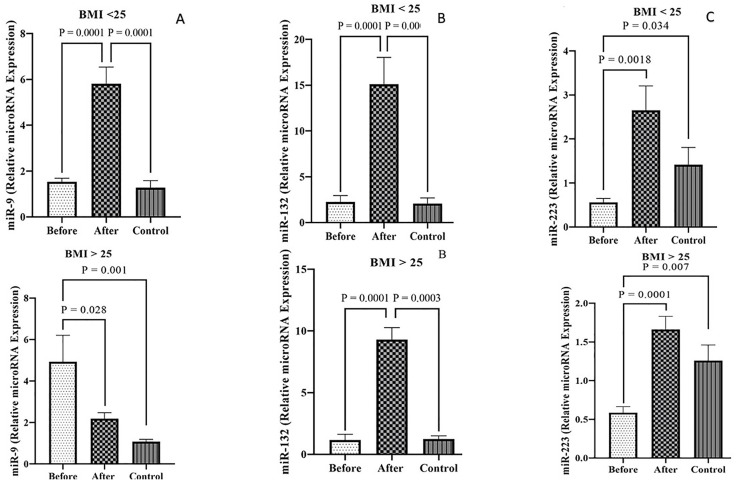
Comparison of the expression levels of *miR-9* (A), *miR-132* (B), and *miR-223* (C) between PCOS patients pre- and post-treatment and control groups with BMI > 25 kg/m² and BMI < 25 kg/m².

**Fig 2:**
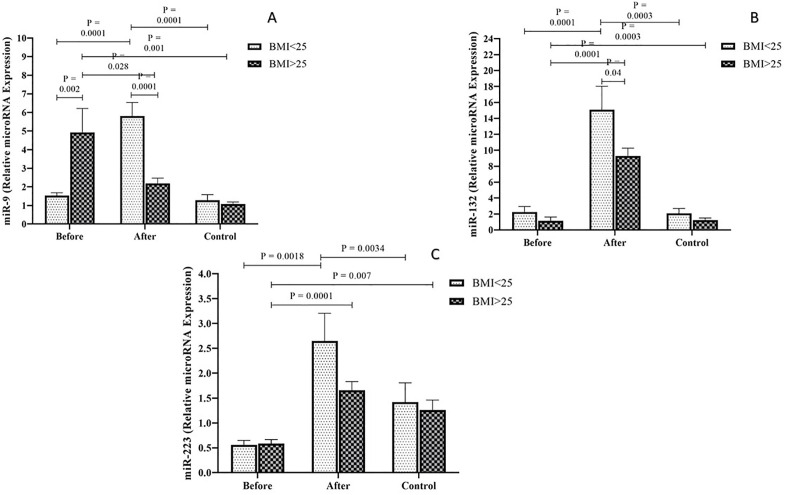
The expression levels of *miR-9* (A), *miR-132* (B), and *miR-223* (C) in obese and non-obese PCOS groups, compared to the control group.

The expression levels of *miR-9* were examined among the PCOS patients and healthy individuals with both BMI > 25 kg/m² and BMI < 25 kg/m² groups. The results indicated a significantly lower expression of *miR-9* among the obese PCOS patients compared to the obese control group (*P* = 0.001). In addition, in the PCOS group with BMI > 25 kg/m², the miR-9 expression levels were significantly higher during Pre-treatment than Post-treatment (P = 0.028). miR-9 expression levels were significantly lower between the non-obese PCOS patients Pre-treatment and non- obese control groups than the PCOS patients one Post-treatment (P = 0.0001) (Fig 1A). Moreover, regarding BMI, the expression level of *miR-9* in pre- treatment PCOS patients with a BMI > 25 kg/m² was significantly higher compared to pre-metformin PCOS patients with a BMI < 25 kg/m², post- treatment PCOS patients with a BMI < 25 kg/m², and the control group with a BMI > 25 kg/m² (P < 0.001) (Fig 2A).

The level of *miR-132* expression was compared among the PCOS and control groups with BMI > 25 kg/m² and BMI < 25 kg/m². Comparison between the *miR-9* expression levels indicated a significantly increase among the PCOS group with both BMI during post- treatment than two other groups (P < 0.001) (Fig 1B). Furthermore, the expression level of *miR-132* in non-obese PCOS patients Post-treatment was significantly higher than that observed in obese PCOS patients Post-treatment, the control group, and non-obese PCOS patients Pre-treatment. Additionally, the expression level of *miR-132* in PCOS patients with a BMI < 25 kg/m² Pre-treatment was found to be lower than that of PCOS patients with a BMI > 25 kg/m² Post-treatment and the control group with a BMI > 25 kg/m² (*P* < 0.001) (Fig 2B).

According to the results, the level of *miR-223* expression in the PCOS groups with BMI > 25 kg/m² and BMI < 25 kg/m² Post-treatment tends to be higher in comparison to the PCOS with BMI > 25 kg/m² and BMI < 25 kg/m² during pre-treatment and control groups (*P* < 0.05) (Fig 1C). The expression level of *miR-223* was notably elevated in non- obese PCOS patients Post-treatment compared to both the non-obese control group and PCOS patients Pre-treatment. Moreover, PCOS patients with a BMI > 25 kg/m² Post-treatment showed higher *miR-223* expression levels than those with a BMI > 25 kg/m² Pre-treatment (*P* < 0.001). Additionally, the findings revealed that *miR-223* expression in the obese control group was greater than that observed in PCOS patients Pre-treatment (*P* < 0.05) (Fig 2C).

### Comparison of the expression level of inflammasome pathway gene expression markers (*IL-1β*, *NLRP3*, *IL-18*, and *Caspase-1*)

**Fig 3:**
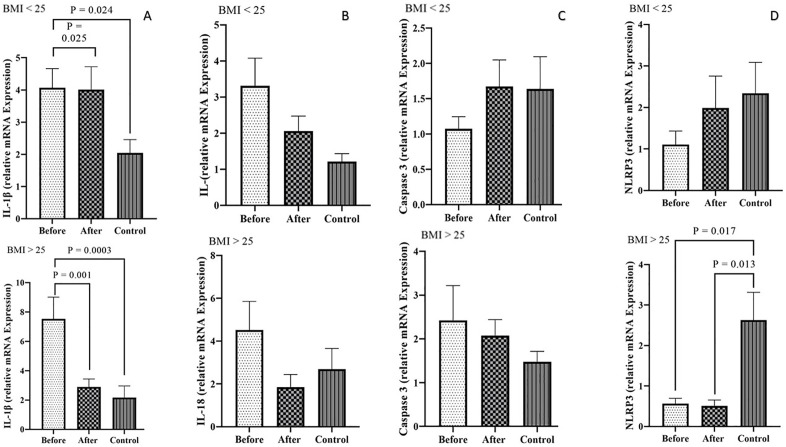
Expression levels of *IL-1β*, *IL-18*, *Caspase-1*, and *NLRP3*among control subjects and PCOS patients before and after metformin treatment, categorized by BMI groups (<25 and >25 kg/m²). Gene expression was normalized to β-actin. Statistical significance was determined using one-way ANOVA (P < 0.05).

**Fig 4:**
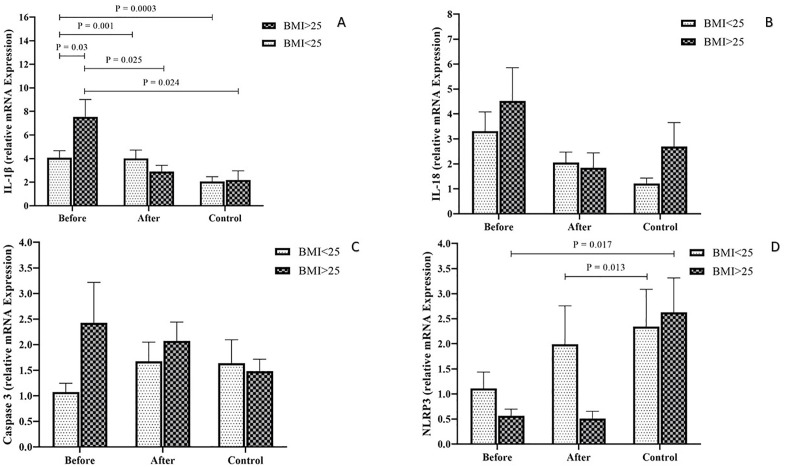
The difference of *IL-1β* (A), *IL-18* (B), *Caspase-1* (C) and *NLRP3* (D) expression between BMI < 25 kg/m² and BMI > 25 kg/m² PCOS groups and the control group.

The results indicated that *IL-1β* expression levels were significantly elevated in both BMI categories within the PCOS group Pre-treatment, compared to the PCOS and control groups in both BMI categories (P < 0.05) (Fig 3A). A comparison of *IL-1β* expression levels among the study groups with varying BMIs revealed that *IL-1β* levels were significantly higher in the PCOS group with a BMI > 25 kg/m² Pre-treatment compared to the Pre-treatment PCOS group with a BMI < 25 kg/m², the PCOS group with a BMI > 25 kg/m² Post-treatment, and the control group with a BMI > 25 kg/m² (*P* < 0.001). Conversely, *IL-1β* expression levels in the PCOS group with a BMI < 25 kg/m² Pre-treatment were significantly lower than those in the Pre-treatment PCOS group with a BMI > 25 kg/m² and the control group with a BMI < 25 kg/m². This reduction in expression levels was statistically significant (*P* < 0.05) (Fig 4A).

The expression level of *IL-18* indicated a reduction in PCOS during post-metformin therapy process and the control groups with BMI > 25 kg/m² and BMI < 25 kg/m² than in the patients with PCOS Pre-treatment, but this alteration was not notable (*P* > 0.05) (Fig 3B). No significant differences were observed in *IL-18* expression levels when comparing the two overweight and obese groups with the control group (*P* > 0.05) (Fig 4B).

In the pre-treatment PCOS group, the level of *Caspase-1* expression could be altered compared with the post-treatment PCOS and control groups with a BMI > 25 kg/m². In contrast, the administration of metformin could increase the *Caspase-1* expression in the PCOS group with BMI < 25 kg/m² in comparison with the two remaining groups. Nevertheless, these modifications were not significant (*P* > 0.05) (Fig 3C). The expression levels of *Caspase-1* showed no significant variation between the two overweight and obese groups and the control group (*P* > 0.05) (Fig 4C).

*NLRP3* expression levels were significantly elevated in the control group with BMI > 25 kg/m² compared to PCOS patients with BMI > 25 kg/m², both pre- and post-treatment (P < 0.05). A similar trend in *NLRP3* expression was observed in healthy women with BMI < 25 kg/m² compared to PCOS patients with BMI > 25 kg/m²; however, this difference was not statistically significant (P > 0.05) (Fig 3D). The expression level of *NLRP3* in the control group with a BMI > 25 kg/m² was significantly higher than that in the PCOS group with a BMI > 25 kg/m² Pre-treatment (*P* = 0.017). Additionally, a comparison of *NLRP3* expression levels between the control group and the PCOS group with a BMI < 25 kg/m² Post-treatment revealed a significantly higher expression in the control group (*P* = 0.013) (Fig 4D).

## Discussion

In this case-control study, we examined the effects of inflammasome-regulating microRNAs (*miR-9*, *miR-223*, and *miR-132*) pre- and post-treatment in obese patients with PCOS, comparing them to healthy individuals. We also assessed the levels of key inflammasome-related genes, including *IL-1β*, *Caspase-1*, *NLRP3*, and *IL-18*. Elevated *miR-9* expression in obese PCOS patients before metformin treatment reflected a heightened pro-inflammatory state, potentially through the NF-κB pathway activation and inflammasome signaling involving IL-1β [35–37]. Following metformin treatment, *miR-9* expression levels significantly decrease, leading to reduced inflammasome activation and cytokine production, highlighting metformin’s anti-inflammatory effects. This suggests that metformin modulates inflammation in obese patients with PCOS, improving both metabolic and inflammatory outcomes, consistent with previous findings on *miR-9* expression in PCOS [37–39]. Moreover, the significant reduction in *miR-9* levels post-treatment highlights its anti-inflammatory effects [37,39,40]. Similar studies have found elevated *miR-9* expression in obese PCOS patients and a reduction after metformin therapy, supporting its potential as a biomarker for disease severity and treatment efficacy [41–45]. However, other research differs by showing no significant change in *miR-9* levels with metformin in obese patients [44] or elevated *miR-9* in obesity alone [46], likely due to variations in study populations, methodologies, and treatment protocols.

The study on *miR-132* expression levels showed that after metformin treatment, there was a significant increase in *miR-132* levels in both BMI groups. Additionally, *miR-132* activates *IL-8* in human pre-adipocytes [47]. Metformin can down regulate the expression of *miR-132* as well [48]. The elevation of *miR-132* during Post-treatment may serve as a compensatory mechanism to balance inflammation as inflammasome activity decreases. Although studies often associate *miR-132* downregulation with reduced inflammation, in PCOS patients, its upregulation might aid in modulating insulin sensitivity and inflammation, complementing metformin’s metabolic and anti-inflammatory effects [49]. However, in the study by Jinget al. the expression of *miR-132* was significantly increased in patients with PCOS [45]. This study found that metformin therapy significantly upregulates *miR-132* expression specifically in non-obese PCOS patients (BMI < 25 kg/m²), with levels exceeding those in obese patients Post-treatment, controls, and their own pre-treatment levels (P < 0.001). These findings suggest a BMI-dependent response to metformin, highlighting *miR-132* as a potential biomarker for treatment efficacy and emphasizing the importance of personalized PCOS management based on BMI [50]. *miR-132* directly inhibits the expression of forkhead box protein A1 (Foxa1) by binding to its 3′ untranslated region (3′UTR) [45]. Foxa1 is a transcription factor that plays a crucial role in various metabolic processes and cell cycle regulation, and its expression is essential for GC viability. In experiments, the downregulation of *miR-132* resulted in significantly increased levels of Foxa1, which was further demonstrated through transfection studies where Foxa1 overexpression reversed the suppressive effects of *miR-132* on GC viability [45,51].

In addition, this study indicated that *miR-223* was upregulated within Post-treatment. Among the known miRNAs, *miR-223* is a potent regulator of some inflammatory responses [52,53]. Furthermore, based on Wang et al. investigation, *miR-223* can be considered as a marker of obesity [54]. *miR-223* serves as a negative regulator of inflammasome activation, and its low expression before treatment may contribute to heightened inflammatory responses in PCOS. Post- treatment, increased *miR-223* levels suppress *NLRP3* activity, reducing inflammasome-driven inflammation and supporting metformin’s anti-inflammatory and metabolic benefits in obese PCOS patients [55,56]. This result was an agreement with research of Udesen et al. that confirmed that level of *miR-223* expression was decreased in the metformin group [57]. This study found that metformin therapy significantly upregulates *miR-223* expression in PCOS patients regardless of BMI, with a more pronounced effect in non-obese individuals who showed higher *miR-223* levels post-treatment compared to controls and their own pre-treatment levels. The significant increase in *miR-223* during Post-treatment suggests that *miR-223* may play a key role in mediating the therapeutic effects of metformin in PCOS. Upregulation of *miR-223* has been detected in the adipose tissue of PCOS patients, and it is positively correlated with insulin resistance (IR) in both PCOS and control subjects [54,58]. Specifically, *miR-223* is known to downregulate GLUT4 expression, which inhibits insulin-stimulated glucose uptake in adipocytes, suggesting its involvement in insulin resistance-related diseases such as type 2 diabetes mellitus (T2DM) and obesity [58]. Furthermore, a significant association has been established between elevated serum levels of *miR-223* and increased obesity prevalence, with odds ratios indicating a higher risk for individuals with lower expression levels [59].

We observed the significant elevation of *IL-1β* expression levels in PCOS patients with both BMI > 25 kg/m² and BMI < 25 kg/m² Pre-treatment suggests a heightened inflammatory state in PCOS, regardless of obesity status *IL-1β*, a key pro-inflammatory cytokine involved in inflammasome activation, plays a significant role in the pathophysiology of PCOS by exacerbating chronic inflammation, insulin resistance, and metabolic dysregulation. Its elevated levels align with findings in conditions of metabolic stress, such as PCOS, obesity, and insulin resistance [19,60]. The study by Nouri et al. showed that the *IL-1β* protein level in serum of COS patients with BMI ≥ 25 was significantly higher than PCOS patient with BMI < 25, but there was no significant difference in non-PCOS individuals with BMI < 25 or ≥25. Therefore, they suggested that based on the obtained results on inflammasome components along with increased expression of *IL-1β* especially in overweight patients, it can be concluded that *IL-18* expression as well as *IL-1β* is probably due to activation of Absent in Melanoma 2 (AIM2), NALP3 or NLR family apoptosis inhibitory protein (NAIP) inflammasome, which may play a critical role in immunopathology of PCOS [19]. The observed differences in results can be explained by the distinction between gene expression and protein translation, as well as the role of obesity. While PCOS increases *IL-1β* gene expression regardless of BMI, obesity intensifies the translation process, resulting in higher IL-1β protein levels in the serum of obese PCOS patients. This demonstrates that the combined impact of PCOS and obesity significantly exacerbates inflammation, unlike in non-PCOS individuals, where BMI has little effect on IL-1β protein levels. Moreover, Obese PCOS patients had significantly higher *IL-1β* levels before metformin treatment compared to non-obese PCOS patients, obese patients Post-treatment, and obese controls. Non-obese PCOS patients before treatment showed lower *IL-1β* levels than both obese PCOS patients prior traetment and non-obese controls. These findings suggest that obesity in PCOS patients is associated with increased *IL-1β* expression and inflammation, and that metformin treatment reduces *IL-1β* levels in obese PCOS patients, highlighting its anti-inflammatory effects. Elevated IL-1β levels contribute to chronic low-grade inflammation common to both obesity and PCOS, linking *IL-1β* to insulin resistance and metabolic dysfunctions in PCOS patients and making the interplay between *IL-1β*, obesity, and PCOS a significant area of research [61].The results indicating a significant increase in *NLRP3* expression in the control group with BMI > 25 kg/m² compared to PCOS patients suggest that the regulation of *NLRP3* may differ between obese individuals with and without PCOS. The heightened expression of *NLRP3* in obese controls might reflect a greater response to obesity-related inflammation, independent of PCOS. In Wang’s study, *NLRP3* and *Caspase-1* expression was significantly higher in GCs from patients with PCOS than in GCs from the control group [62]. In addition, In the study of Amer et al., they showed that obesity and PCOS seem to be associated with upregulated expression of *NLRP3* inflammasome components [63]. The reason for the difference between these results and the result of our study is the difference in the type of samples used and study. This study found that *NLRP3* expression levels were consistently lower in PCOS patients compared to healthy controls across various BMI categories, both pre and post treatment. This is unexpected because *NLRP3* plays a key role in mediating inflammatory responses, and since PCOS is associated with chronic low-grade inflammation, higher NLRP3 levels would typically be anticipated in PCOS patients rather than reduced levels. Studies have demonstrated that high-fat diets induce *NLRP3* activation, which contributes to obesity and associated metabolic disorders [62,64]. Specifically, the activation of *NLRP3* is influenced by saturated fatty acids, which are prevalent in obesogenic diets and can exacerbate inflammatory processes [63,65].

The observed reduction in *IL-18* expression levels in the PCOS group Post-treatment, although not statistically significant, is consistent with the known anti-inflammatory effects of metformin. *IL-18*, similar to *IL-1β*, is an inflammasome-mediated cytokine that contributes to systemic inflammation [66]. The downward trend in *IL-18* post-treatment could suggest that metformin helps to partially alleviate inflammasome-driven inflammation in PCOS, which has also been supported by other research showing metformin’s ability to modulate *IL-18* levels in conditions of insulin resistance and metabolic dysfunction [19]. However, the lack of a significant change might indicate that the duration of metformin therapy or the dosage used in the study was insufficient to produce a marked impact on *IL-18* levels, suggesting the need for longer-term interventions to observe significant changes. This result was a disagreement with some investigations. Their results indicated that adipose tissue *IL-18* gene expression levels were significantly higher in obese versus non-obese individuals [19,67,68], while in other study there was no significant difference in *IL-18* levels between the two groups [69]. In another study, the serum *IL-18* concentration in PCOS-like mice was significantly higher than that in control mice [62]. These different results may be due to the participants entered into studies.

In the case of *Caspase-1*, its higher expression in the PCOS group Pre-treatment is consistent with the elevated inflammasome activity in untreated PCOS. *Caspase-1* is crucial for the maturation and secretion of IL-1β and IL-18, making its elevated expression a marker of active inflammasome signaling [70]. Increased *Caspase-1* expression was unexpectedly observed in the PCOS group with BMI < 25 kg/m² Post-treatment, though not significantly. This may indicate a compensatory response or a shift in inflammation dynamics, contrasting with other studies that report reduced *Caspase-1* activity with metformin. Such discrepancies could arise from differences in patient populations, metformin dosage, or participants’ metabolic status. Additionally, Wang and colleagues found significantly higher *Caspase-1* levels in GCs from PCOS patients compared to controls, with similar findings in a mouse model of polycystic ovarian changes [62]. In addition, Guo et al. reported higher levels of mRNA expression levels of *Caspase-1* PCOS versus control women. Moreover, the results showed that 12-week treatment with metformin, resulted in a reduction in *Caspase-1* [67]. The incongruent results obtained in different studies could be attributed to various factors, such as sample sizes, varying participant characteristics, in addition to different sample utilized.

Metformin treatment in PCOS patients has been associated with reduced FSH levels, suggesting it may help regulate hormonal imbalances. FSH plays a crucial role in ovarian function, often disrupted in PCOS due to issues with the hypothalamic-pituitary-ovarian (HPO) axis. This reduction in FSH may result from metformin improving insulin sensitivity and reducing hyperinsulinemia, which affects gonadotropin secretion. Studies show metformin can modulate reproductive hormone levels, enhancing ovulatory function. While Guzel et al. found a decrease in FSH levels with metformin treatment, Dehghan-Kooshkghazi et al. did not observe such changes in obese PCOS patients. Differences in these results may stem from variations in patient demographics and treatment protocols [71]. On the other hand, Dehghan-Kooshkghazi et al. could not illustrate that metformin can change the expression level of FSH among obese PCOS patient [72]. The reason for the difference between this result and the result of our study is the difference in the demographic characteristics of the participating patients and duration and dosage of metformin treatment.

The significant elevation of LH levels in the PCOS group Pre-treatment compared to the treated PCOS group and controls (P < 0.05) aligns with the characteristic hormonal profile of PCOS, where elevated LH is associated with hyperandrogenism and disrupted ovarian function. Metformin’s ability to lower LH levels post-treatment suggests that it may help normalize the elevated LH-to-FSH ratio seen in PCOS, which is a common marker of the syndrome. This reduction in LH after metformin therapy has been documented in other studies, where metformin was shown to reduce LH levels, leading to improvements in menstrual regularity and ovulatory cycles in PCOS patients. Previous studies indicated that metformin was effective in decreasing LH [73]. However, Messinis et al. did not observe a significant change in the LH levels through Post-treatment [74]. A variety of factors, including size of the sample and characteristics of the participants, could explain the inconsistency of results found in different studies.

In the present study, T3 and T4 ratios were significantly lower in the control group compared to PCOS patients, both pre and post treatment (P < 0.001), suggesting thyroid dysfunction may be more common in PCOS patients. While thyroid function is not directly affected by metformin, PCOS can influence thyroid hormone levels through interactions with insulin resistance. This aligns with findings that link PCOS to subclinical hypothyroidism [75,76]. Ibraheem et al. reported a non-significant increase in T3 and T4 levels post- treatment, while TSH significantly decreased [77]. Variations in ageand participant characteristics can lead to differing outcomes.

The absence of significant changes in FBS and TSH levels between the three studied groups (p > 0.05) suggests that metformin’s primary effects on glucose regulation may not be as pronounced when comparing treated and untreated PCOS patients in this specific cohort. Metformin is well-known for its role in improving glucose metabolism [78], but the lack of significant differences here could be due to variations in baseline insulin sensitivity. Dehghan-Kooshkghazi et al. reported that metformin therapy could result in a significant decrease in total testosterone levels and FBS [72]. This difference in results may result from differences in patient characteristics, such as baseline insulin resistance, as well as variations in study design, duration, and dosage of metformin treatment.

In the present study, the stable TSH levels across the groups, despite differences in thyroid hormones (T3 and T4), further indicate that metformin’s impact on thyroid function may be limited or secondary to its primary metabolic effects [79]. Similarly, Hirschberg et al. concuded that the TSH level was not affected by metformin [80]. A meta-analysis study by Di Minno et al. reported that metformin can induce a reduction in TSH levels [81]. A potential explanation to the discrepant results could be potential effects of metformin on thyroid function in PCOS might be subtle or dependent on individual variations in thyroid function and metabolic status.

Regarding limitations of this study, relatively small sample size and lack of long-term follow-up to assess sustained miRNA expression changes might be considere in the future investigations.

## Conclusion

In conclusion, this study highlights the significant role of metformin in modulating the expression of inflammasome-regulating miRNAs (*miR-9*, *miR-223*, and *miR-132*) and related genes in obese PCOS patients. Metformin effectively reduces *miR-9* and *IL-1β* levels, reflecting its anti-inflammatory properties, while elevating *miR-132* and *miR-223* levels, which may contribute to improved insulin sensitivity and inflammation control. These findings underscore the therapeutic potential of metformin in addressing both metabolic and inflammatory imbalances in PCOS, particularly in obese individuals. However, further research is needed to explore long-term effects and optimize treatment protocols.
